# Oxidative Stress, NF-κB-Mediated Inflammation and Apoptosis in the Testes of Streptozotocin–Induced Diabetic Rats: Combined Protective Effects of Malaysian Propolis and Metformin

**DOI:** 10.3390/antiox8100465

**Published:** 2019-10-09

**Authors:** Victor Udo Nna, Ainul Bahiyah Abu Bakar, Azlina Ahmad, Chinedum Ogbonnaya Eleazu, Mahaneem Mohamed

**Affiliations:** 1 Department of Physiology, School of Medical Sciences, Universiti Sains Malaysia, Kubang Kerian 16150, Kelantan, Malaysia; victorudon@unical.edu.ng (V.U.N.); ainul@usm.my (A.B.A.B.); chinedum.eleazu@funai.edu.ng (C.O.E.); 2 Department of Physiology, Faculty of Basic Medical Sciences, College of Medical Sciences, University of Calabar, Calabar P.M.B. 1115, Cross River State, Nigeria; 3 Basic Science and Oral Biology Unit, School of Dental Sciences, Universiti Sains Malaysia, Kubang Kerian 16150, Kelantan, Malaysia; azlinakb@usm.my; 4 Department of Chemistry/Biochemistry/Molecular Biology, Federal University Ndufu Alaike Ikwo, Abakiliki P.M.B. 10, Ebonyi State, Nigeria; 5 Unit of Integrative Medicine, School of Medical Sciences, Universiti Sains Malaysia, Kubang Kerian 16150, Kelantan, Malaysia

**Keywords:** antioxidant, apoptosis, diabetes, inflammation, Malaysian propolis, metformin

## Abstract

Oxidative stress, inflammation and apoptosis are major complications that trigger organ failure in diabetes mellitus (DM), and are proven to adversely affect the male reproductive system. Clinical and experimental studies have demonstrated the promising protective effects of propolis in DM and its associated systemic effects. Herein, we investigated the effect of Malaysian propolis (MP) on testicular oxidative stress, inflammation and apoptosis in diabetic rats. Further, the possibility of a complementary effect of MP with the anti-hyperglycaemic agent, metformin (Met), was studied with the idea of recommending its use in the event that Met alone is unable to contain the negative effects of DM on the male reproductive system in mind. Male Sprague-Dawley rats were either gavaged distilled water (normoglycaemic control and diabetic control groups), MP (diabetic rats on MP), Met (diabetic rats on Met) or MP+Met (diabetic rats on MP+Met), for 4 weeks. MP decreased oxidative stress by up-regulating (*p* < 0.05) testicular mRNA levels of nuclear factor erythroid 2-related factor 2, superoxide dismutase, catalase and glutathione peroxidase; increasing (*p* < 0.05) the activities of antioxidant enzymes; and decreasing (*p* < 0.05) lipid peroxidation in the testes and epididymis of diabetic rats. Further, MP down-regulated (*p* < 0.05) testicular mRNA and protein levels of pro-inflammatory mediators (nuclear factor kappa B, inducible nitric oxide synthase, tumour necrosis factor-α and interleukin (IL)-1β), decreased (*p* < 0.05) the nitric oxide level, and increased (*p* < 0.05) IL-10 mRNA and protein levels. MP also down-regulated (*p* < 0.05) Bax/Bcl-2, p53, casapase-8, caspase-9 and caspase-3 genes, and increased (*p* < 0.05) testicular germ cell proliferation. MP’s effects were comparable to Met. However, the best results were achieved following co-administration of MP and Met. Therefore, we concluded that administration of the MP+Met combination better attenuates testicular oxidative stress, inflammation and apoptosis in DM, relative to MP or Met monotherapy, and may improve the fertility of males with DM.

## 1. Introduction

Diabetes mellitus (DM) has been reported to affect the male gonad negatively, with a decrease in fertility rate [1,2]. Mounting evidence from both clinical and experimental studies have demonstrated decreased fertility rates, and have linked the same with testicular oxidative stress, inflammation and apoptosis [3,4,5]. In fact, in our previous studies, we found that not only did DM increase testicular oxidative stress [6], but it also increased oxidative stress in the cauda epididymis [2], thus exposing the stored spermatozoa to further risk of oxidative stress. Particularly, down-regulation of nuclear factor erythroid 2-related factor 2/antioxidant response elements (Nrf2/ARE) and up-regulation of nuclear factor kappa B (NF-κB)-mediated inflammation have been reported in the testes of diabetic rats [3,6,7,8].

Oxidative stress and inflammation are common features of DM, and are reportedly responsible for the multi-organ complications associated with the disease. Both conditions reportedly share the same activation stimulus, reactive oxygen species (ROS), which are produced in large concentrations as a result of the increase in glucose auto-oxidation in DM [9]. Furthermore, DM-mediated oxidative stress and inflammation can trigger apoptotic signals, leading to cellular/tissue damage and resultant organ failure. Therefore, the use of antioxidants as complementary medicines to DM treatment becomes pertinent, in other to curb its ROS-associated complications.

Propolis is a gluey mixture of resinous substances which are deployed by honey bees to repair broken hives. Propolis contains sap, exudates from tree and leaf buds, and wax [10]. Generally, propolis is reported to contain 50% resin, 30% wax, 10% essential and aromatic oils, 5% pollen and 5% other organic substances [11,12]. To date, over 300 chemical components have been identified in propolis, and studies have concluded that the chemical composition of propolis may differ depending on its geographical region and bee species [10,13].

Propolis from many regions have been chemically characterised. The main components common to propolis from Turkey, Greece, the United States of America, Malaysia, Thailand, Australia, China, Brazil, Bulgaria, Algeria, Egypt, Cameroon and Congo, are flavonoids, aromatic acids, phenolic compounds, and esters of caffeic and ferulic acids [14,15,16,17,18]. Some biological activities have been ascribed to propolis, and they include: anti-hyperglycaemic, antioxidant, anti-inflammatory, anti-apoptotic and anti-microbial effects [13,19,20]. Others include renoprotective [21,22], cardioprotective [23,24] and hepatoprotective [25,26] effects.

Clinical trials of propolis on diabetic patients have yielded conflicting results [27,28,29,30]. However, most studies have demonstrated significant decreases in blood glucose, oxidative stress and inflammatory biomarkers [27,28,29], which are consistent with animal studies [20]. There is a need, therefore, to study the complementary effects of propolis with established anti-hyperglycaemic agents as a step towards its use as a complementary therapy in the event that existing medications fail to achieve normoglycaemia, which however, is often the case.

We previously demonstrated significant α-glucosidase inhibition and antioxidant activities of Malaysian propolis (MP) in vitro [20]. We also demonstrated a significant decrease in fasting blood glucose and an increase in insulin levels in rats treated with MP + metformin (Met). Worthy of noting is that the combined therapy presented better results relative to the monotherapy-treated (MP or Met) rats. Herein, we attempt to probe MP’s effect on DM-induced testicular oxidative stress, inflammation and apoptosis, in a bid to find answers regarding its ability to protect the male reproductive system from oxidative damage, inflammation and apoptosis in a diabetic state. Further, its possible complementary effects with the anti-hyperglycaemic medication, Met, was assessed with a view to recommend its use should Met alone fail to contain the negative effects of DM on the male reproductive system.

## 2. Materials and Methods

### 2.1. Chemicals

Streptozotocin (STZ), agarose and RNAlater were procured from Sigma-Aldrich Chemical Company (St, Louis, MO, USA), while metformin was procured from Hovid Bhd, Malaysia. Rabbit polyclonal primary antibodies for nuclear factor kappa B (NF-κB) (PAB824Ra01), tumour necrosis factor alpha (TNF-α) (PAA133Ra01), interleukin 1β (IL-1β) (PAA563Ra01), IL-10 (PAA056Ra01), Caspase-3 (PAA626Ra01) and proliferating cell nuclear antigen (PCNA) (PAA591Mi01) were procured from Cloud-Clone Corp (Katy, TX USA), while Dako EnVision™+ System/HRP, Rb (DAB+) for signal detection was procured from Agilent Technologies, Inc. (Santa Clara, CA USA). Innu Prep RNA mini kit was supplied by Analytik Jena AG (Jena, Germany), SensiFAST SYBR Hi-ROX One-Step PCR kit was procured from Bioline and primers were supplied by Integrated DNA Technologies (IDT, Pulau Pinang, Malaysia). PCR tubes and strips were sourced from Applied Biosystems (Life technologies, Shanghai, China). All other chemicals that were used for this study and which were not listed above, were of analytical grade.

### 2.2. Extraction of Propolis

Malaysian propolis (MP) *itama*, from the stingless bees *Heterotrigona itama*, were sourced from a bee farm in Kelantan, Malaysia. MP was extracted in 70% ethanol as previously described in detail by our group [20] and stored at −20 °C until use. We previously reported that this MP extract showed inhibition of α-glucosidase activity and possessed antioxidant activity (as determined using 2,2-diphenyl-1-picryl-hydrazyl-hydrate (DPPH) radical, ferric reducing ability of plasma (FRAP) and hydrogen peroxide (H_2_O_2_) scavenging activities in vitro [20]). Further, we reported the presence of large amounts of flavonoid and phenolic compounds, and identified coumaric and gallic acid derivatives, ellagic acid and resveratrol, using liquid chromatography–mass spectrometry analysis [31]. We also identified the presence of ethyloctadecanoate, hexadecanoic acid, and 8 and 9-octadecenoic acids, using gas chromatography–mass spectrometry analysis [32].

### 2.3. Laboratory Animals

Forty adult male Sprague-Dawley rats weighing 250–300 g were utilised for the present study. They were sourced from the Animal Research and Service Centre, Universiti Sains Malaysia (USM), Kelantan, Malaysia, and housed in the Animal House, Department of Physiology, School of Medical Sciences, USM. The rats were maintained in a 12/12 h reversed light/dark cycle, with lights on at 12:00 and off at 00:00. The rats were allowed ad libitum access to a standard pellet diet and drinking water. The rats were acclimatised in these conditions for 7 days before commencement of the experiment. This study protocol was approved by the Animal Ethics Committee of USM, with the following approval number: USM/IACUC/2017/(831). All rats were handled in compliance with the Principles for the Care and Use of Laboratory Animals by the National Institute of Health.

### 2.4. Induction of Diabetes Mellitus

Diabetes was induced in 32 randomly selected rats as described previously by our group [20]. Briefly, a single dose of STZ (60 mg/kg body weight (b.w.)) dissolved in ice-cold saline was injected intraperitoneally, after an overnight fast. The normoglycaemic control group (*n* = 8) was injected with 1 mL of ice-cold normal saline. The drinking water of STZ-injected rats was replaced with 5% glucose, which the rats drank ad libitum overnight on day 1, to prevent hypoglycaemia and mortality. A fasting blood glucose (FBG) level ≥ 250 mg/dL measured 72 h post-STZ injection, as recorded using a glucometer (URight TD-4279 Blood Glucose Monitoring System, Munster, Germany), confirmed successful DM induction [20].

### 2.5. Experimental Design

The rats were randomised into 5 groups (*n* = 8/group) as follows: normoglycaemic control (NC), diabetic control (DC), diabetic on 300 mg/kg b.w./day of MP (D+MP), diabetic on 300 mg/kg b.w./day of metformin (D+Met) and diabetic on MP+Met (D+MP+Met). MP and Met were each suspended in 1 mL of distilled water before oral administration at 09:00 for 4 weeks, while rats in NC and DC groups were gavaged distilled water during the same period. The selected doses for MP and Met were based on our previous investigation [20]. At the end of the treatment period, the rats were fasted overnight, and euthanised under pentobarbital anaesthesia (60 mg/kg b.w.), which was administered intraperitoneally.

### 2.6. Weight and Relative Weights of Reproductive Organs

The rats were weighed, euthanised, and their reproductive organs (testes, epididymis, seminal vesicle and prostate) were excised and weighed. Thereafter, the relative organ weights were calculated as follows:

Relative weight (%) = (Organ weight (g)/Final body weight (g)) × 100

### 2.7. Sample Collection and Preparation

The left testis and cauda epididymis were cleared of surrounding tissues and rinsed in ice-cold saline. From the testis, one portion was immediately kept in RNAlater and stored at −80 °C pending usage, while the other portion and the cauda epididymis were separately used to prepare a 10% (*w/v*) homogenate in ice-cold Tris-HCl buffer (pH 7.4), centrifuged at 1000× *g* for 20 min using a refrigerated centrifuge. The supernatant was obtained and stored at −80 °C until use. For glutathione (GSH) assay, 100 µL of the samples (supernatant) were deproteinized [33]. Briefly, 100 µL of each sample was mixed with 100 µL of 10% (*w/v*) metaphosphoric acid. The content was vortexed for 10 s and allowed to stand at room temperature for 5 min, followed by centrifugation at 1200× *g* for 15 min at 4 °C. A 100 µL aliquot of the resulting supernatant was pipetted into a separate 1.5 mL tube, followed by the addition of 5 µL of 4 M triethanolamine solution, and vortexed to mix. The samples were kept at −80 °C until use.

### 2.8. Histopathology of the Testes and Epididymis

The right testis and cauda epididymis were placed in Bouin’s solution for 24 h, dehydrated, and embedded in paraffin blocks. From each tissue block, 5 µm thick sections were stained using haematoxylin and eosin (H&E). Testicular tissue histological sections were observed under a light microscope (Olympus BX41, Olympus Corporation, Tokyo, Japan). Leydig cells were counted in 20 intertubular spaces per rat. For each rat, 20 round seminiferous tubules were randomly selected, and their diameters and epithelial heights were measured at ×100 magnification using Image Analyser software (Soft Imaging System, VGA Utilities version 3.67e, Tokyo, Japan). Further, we examined 200 seminiferous tubules for assessment on germ cells loss (either focal or complete) using the ×10 objective, and the result was expressed as a percentage of the total number (200) of seminiferous tubules examined [2,6].

### 2.9. Testicular and Epididymal Oxidative Stress Status

Testicular and epididymal antioxidant/oxidative stress markers were assessed using their respective supernatants following well established methods. The activity of SOD was determined using the nitro tetrazolium blue reduction method [34]. CAT activity was determined using molybdate reaction with hydrogen peroxide [35]. The activity of GPx was determined using glutathione oxidation method, with hydrogen peroxide as substrate [36]. GST activity was determined using the GSH conjugation method, with 1-chloro-2,4-dinitrobenzene as substrate [37], whereas GR activity was assessed using glutathione disulphide reduction method [38]. Total GSH level was determined as described by Annuk et al. [33]. Thiobarbituric acid reactive substance (TBARS) level was determined using the method of Ohkawa et al. [39], while TAC was determined using the method of Koracevic et al. [40]. Testicular nitric oxide (NO) level was assessed using a commercially available ELISA kit (BioAssay Systems, Hayward, CA, USA). The results of the antioxidant activities were normalised with the protein level of each sample, which was measured using a total protein assay kit (BioAssay Systems, Hayward, CA, USA).

### 2.10. Testicular mRNA Transcript Levels of Antioxidant, Inflammation and Apoptosis-Related Genes

#### 2.10.1. RNA Extraction, Quality and Purity Determination

Each testis tissue was thawed, removed from RNAlater and pad-dried. Thereafter, RNA was extracted from each tissue using Innu Prep RNA mini kit (Analytik Jena, Jena, Germany), following the manufacturer’s protocol. The concentration and purity of extracted RNA was determined using a Nanodrop spectrophotometer (Eppendorf Nanodrop BioPhotometer plus, Hamburg, Germany. To determine RNA quality, gel electrophoresis using 1% agarose (*w/v*) in 1 × LB buffer, was performed and viewed using a UV transilluminator (ChemiDoc XRS, Bio-Rad Laboratories, Hercules, CA, USA). RNA samples which had OD_260/280_ of 1.8–2.0 as determined by a Nanodrop spectrophotometer, and showed distinct 28S and 18S ribosomal RNA bands after gel electrophoresis, were selected for the downstream experiments.

#### 2.10.2. Real Time Reverse Transcription-Quantitative Polymerase Chain Reaction (RT-qPCR)

SensiFAST SYBR Hi-Rox One-Step PCR kit (Bioline, London, UK) was used for PCR amplification, following the manufacturer’s protocol. This kit employs a single step from first-strand cDNA synthesis to real-time PCR amplification in a single reaction tube. Real time RT-qPCR was performed using StepOnePlus Real-Time PCR system (Applied Biosystems Co., Foster City, CA, USA). The forward and reverse primers employed for the present study were selected from GenBank (Table S1) and synthesised by Integrated DNA Technologies (IDT, Bayan Lepas, Malaysia). For each target, the melting curve and standard curve experiments were performed to validate primer specificity and efficiencies, which were >90%. For each target, 3-step cycling was performed and included: initial denaturation for 2 min at 95 °C, 40 cycles of (i) denaturation for 5 s at 95 °C, (ii) annealing for 10 s at 60 °C and (iii) extension for 5 s at 72 °C. All samples were analysed in triplicate and results were normalised using *GAPDH* as the housekeeping gene. For relative quantification, 2^−ΔΔCt^ method was employed [41].

### 2.11. Immunohistochemical Study in the Testes

Five micrometre thick testis sections were employed for immunohistochemistry as per our previously described protocol [6]. Heat-mediated antigen retrieval was performed for 5 min, using a pressure cooker containing Tris-EDTA buffer with 0.05% tween 20 (pH 9.0). Thereafter, endogenous peroxidase was blocked using 3% hydrogen peroxide in phosphate buffered saline for 5 min, rinsed with distilled water and Tris-buffered saline containing 0.05% tween 20 (TBST, pH 8.4). Testes sections were incubated with polyclonal primary antibodies for NF-kB(p65) (1:80), IL-1β (1:80), IL-10 (1:40), caspase-3 (1:150) and PCNA (1:50) (Cloud-Clone Corp, Houston, TX, USA) overnight at 4 °C, while incubation with TNF-α (1:120) polyclonal primary antibody was performed for 1 h at room temperature. Sections were washed twice with TBST for 5 min, followed by incubation with Dako EnVision™+ System/HRP labelled polymer containing goat anti-rabbit secondary antibody (Agilent Technologies, Inc. Santa Clara, USA) for 30 min, at room temperature. Dako 3,3′-diaminobenzidine substrate (Agilent Technologies, Inc. Santa Clara, USA) was used for detection. Haematoxylin was used for counter staining, followed by dehydration and viewing using a light microscope (Olympus BX41, Olympus Corporation, Tokyo, Japan). The intensity of brown staining was quantified using ImageJ software (ImageJ, NIH-Bethesda, MD, USA) and the results for the test groups were expressed as fold changes in intensity relative to NC group.

### 2.12. Statistical Analysis

Data obtained from the present study were analysed using GraphPad Prism 7.0 (GraphPad Software Inc., La Jolla, CA, USA), and results are presented as mean ± standard deviation (SD). The distribution and variance of the data sets were assessed using Shapiro–Wilk and D’Agostino–Pearson Omnibus normality tests, respectively, before employing one-way analysis of variance (ANOVA), followed by Tukey’s post-hoc test. Values of *p* < 0.05 were considered statistically significant.

## 3. Results

### 3.1. Blood Glucose Level

**Figure 1:**
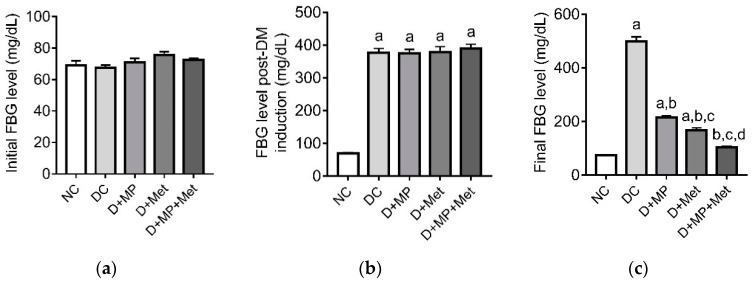
Initial fasting blood glucose level (**a**), fasting blood glucose level post-diabetes induction (**b**) and final fasting blood glucose level (**c**). Values are means ± SDs, *n* = 8. ^a^ *p* < 0.05 versus NC, ^b^ *p* < 0.05 versus DC, ^c^ *p* < 0.05 versus D+MP, ^d^ *p* < 0.05 versus D+Met (one-way ANOVA followed by Tukey’s post-hoc test). NC: normoglycaemic control, DC: diabetic control, D+MP: diabetic rats treated with Malaysian propolis, D+Met: diabetic rats treated with metformin, D+MP+Met: diabetic rats treated with Malaysian propolis + metformin.

The initial FBG level did not differ across the groups, suggestive of the fact that the rats were normoglycaemic at the start of the experiment (Figure 1a). Forty-eight hours post-DM induction, the FBG levels in DC, D+MP, D+Met and D+MP+Met groups increased (*p* < 0.05) relative to NC group (Figure 1b), indicating successful DM induction. At the end of the treatment period, the final FBG levels remained higher (*p* < 0.05) in DC, D+MP and D+Met groups, relative to NC group, but were lower (*p* < 0.05) in all treatment groups, relative to DC group (Figure 1c). The final FBG level decreased (*p* < 0.05) in D+Met group, relative to D+MP group. Noteworthy, is that the final FBG level in the combined therapy group decreased (*p* < 0.05) relative to the monotherapy groups, and was comparable to NC group (Figure 1c).

### 3.2. Weights and Relative Weights of Reproductive Organs

The absolute weight of the testes decreased (*p* < 0.05) in DC and D+Met groups, relative to NC group, but increased (*p* < 0.05) in all treatment groups, relative to DC group. The relative testes’ weights decreased (*p* < 0.05) in DC group relative to NC group, but increased (*p* < 0.05) in D+MP group relative to DC group (Table 1). The absolute epididymal weight decreased (*p* < 0.05) in DC, D+MP and D+Met groups, relative to NC group, but increased (*p* < 0.05) in all the treatment groups, relative to DC group, and increased (*p* < 0.05) in D+MP+Met group relative to D+Met group. However, the relative epididymal weight did not differ (*p* > 0.05) between the groups (Table 1). The absolute and relative weights of prostate gland decreased (*p* < 0.05) in DC and D+Met groups, relative to NC group, but increased (*p* < 0.05) in D+MP and D+MP+Met groups relative to DC group, and increased (*p* < 0.05) in D+MP+Met group relative to D+Met group (Table 1). The absolute and relative seminal vesicle weights decreased (*p* < 0.05) in all groups except D+MP+Met group, relative to NC group. Meanwhile, the absolute and relative seminal vesicle weights increased (*p* < 0.05) in all treatment groups, relative to DC group, and increased (*p* < 0.05) in the combined treatment group relative to the monotherapy groups (Table 1).

### 3.3. Histopathology of the Testes and Epididymis

**Figure 2:**
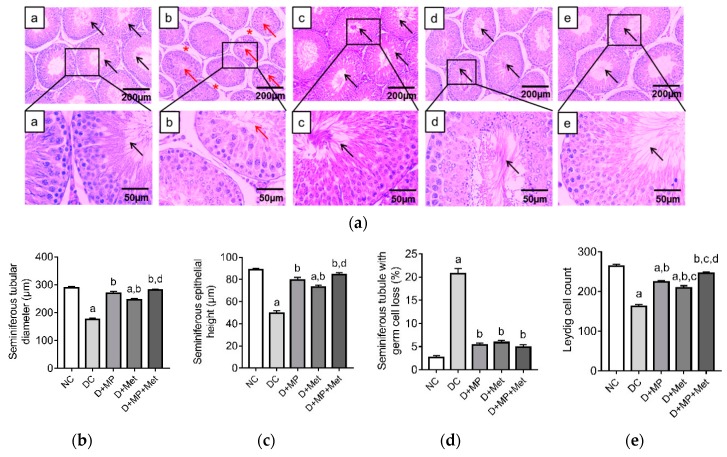
Representative photomicrographs of the testes in (a) NC, (b) DC, (c) D+MP, (d) D+Met and (e) D+MP+Met groups (**a**). The testes histology for DC group showed numerous seminiferous tubules with incomplete spermatogenesis (red arrows), and large intertubular spaces (red asterisk), relative to NC and the treated groups, which showed numerous seminiferous tubules with complete spermatogenesis (black arrows) (haematoxylin and eosin staining; magnification: ×100 and ×400; scale bars: 200 µm and 50 µm, for upper and lower panels, respectively). For (**b**) seminiferous tubular diameter, (**c**) seminiferous epithelial height, (**d**) seminiferous tubule with germ cell loss and (**e**) Leydig cell count, values are mean ± SD, *n* = 8. ^a^ *p* < 0.05 versus NC, ^b^ *p* < 0.05 versus DC, ^c^ *p* < 0.05 versus D+MP, ^d^ *p* < 0.05 versus D+Met (one-way ANOVA followed by Tukey’s post-hoc test). NC: normoglycaemic control, DC: diabetic control, D+MP: diabetic rats treated with Malaysian propolis, D+Met: diabetic rats treated with metformin, D+MP+Met: diabetic rats treated with Malaysian propolis + metformin.

Generally, the seminiferous tubules in the testes of DC group had decreased diameters and epithelial heights. As a result, there were large inter-tubular spaces between adjacent seminiferous tubules. Many seminiferous tubules in DC group had lost a considerable amount of germ cells and had few or no spermatozoa in the seminiferous tubular lumen (Figure 2a). The testes’ histological structures were improved in the treatment groups, with the combined treatment group showing the best improvements (Figure 2a). Quantitative data obtained from the photomicrographs showed decreases (*p* < 0.05) in seminiferous tubular diameter, seminiferous epithelial height and Leydig cell count, and an increase (*p* < 0.05) in the percentage of seminiferous tubules with germ cell loss in DC group, relative to NC group (Figure 2b–e). Similarly, seminiferous tubular diameter and epithelial height decreased (*p* < 0.05) in the D+Met group, while Leydig cell count decreased (*p* < 0.05) in D+MP and D+Met groups, relative to NC group. Nevertheless, these parameters were improved (*p* < 0.05) in all treatment groups, relative to DC group (Figure 2b–e). Furthermore, MP exerted comparable effects with Met on seminiferous tubular diameter, epithelial height and the percentage of tubules with germ cell loss, but MP increased Leydig cell count (*p* < 0.05) relative to Met. Increases in seminiferous tubular diameter, epithelial height and Leydig cell count in the combined therapy group were significant relative to D+Met group (Figure 2b–e).

**Figure 3:**
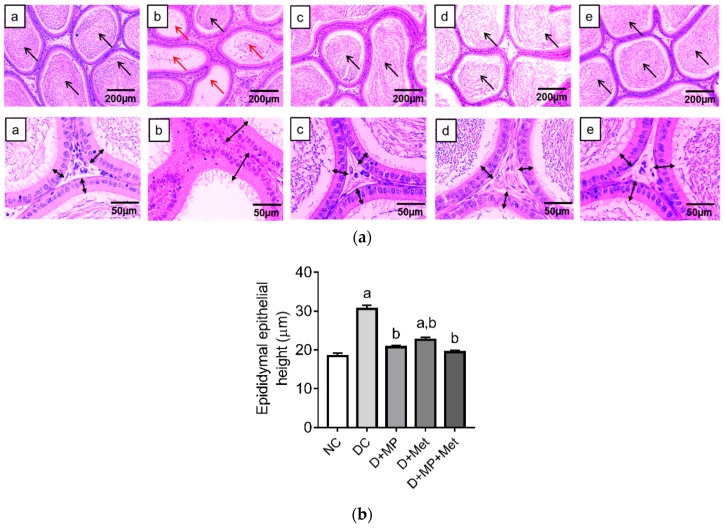
Representative photomicrographs of the caudal epididymis in (a) NC, (b) DC, (c) D+MP, (d) D+Met and (e) D+MP+Met groups (**a**). Histology of the epididymis of DC group showed large areas with decreased sperm density (red arrows) and increased epithelial height, relative to the NC group and treated groups (c,d,e) which showed large areas with high sperm density (black arrows) (haematoxylin and eosin staining, magnification: ×100 and ×400; scale bar: 200 µm and 50 µm for upper and lower panels, respectively). For (**b**) epithelial height, values are mean ± SD, *n* = 8. ^a^ *p* < 0.05 versus NC, ^b^ *p* < 0.05 versus DC (one-way ANOVA followed by Tukey’s post-hoc test). NC: normoglycaemic control, DC: diabetic control, D+MP: diabetic rats treated with Malaysian propolis, D+Met: diabetic rats treated with metformin, D+MP+Met: diabetic rats treated with Malaysian propolis + metformin.

Histology of the cauda epididymis showed numerous areas with decreased sperm density in DC group, relative to NC group, which showed numerous areas with high sperm density. In the treatment groups, epididymal histology was comparable to the NC group, with numerous areas having high sperm density (Figure 3a). At a higher magnification (×400), thickening of the epithelium of the cauda epididymis was seen in DC group compared to NC group, while the epithelial layer was less thickened in the treatment groups relative to DC group (Figure 3a). Quantitative data showed an increase (*p* < 0.05) in cauda epididymal epithelial height in DC and D+Met groups compared to NC group (Figure 3b). However, the epididymal epithelial height decreased (*p* < 0.05) in all treatment groups compared to DC group (Figure 3b).

### 3.4. mRNA Transcript Levels of Antioxidant-Related Genes

**Figure 4:**
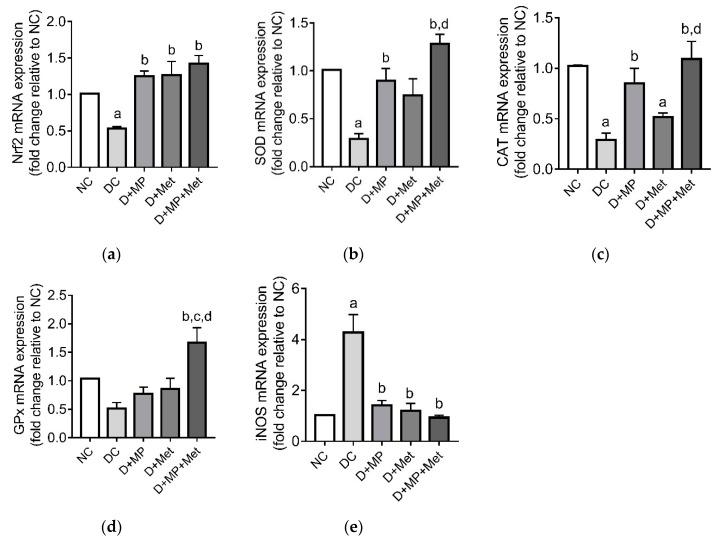
The effect of Malaysian propolis, metformin and their combination on testicular mRNA expressions of (**a**) Nrf2, (**b**) SOD, (**c**) CAT, (**d**) GPx and (**e**) iNOS in diabetic rats. Values are mean ± SD, *n* = 6. ^a^ *p* < 0.05 versus NC, ^b^ *p* < 0.05 versus DC, ^c^ *p* < 0.05 versus D+MP, ^d^ *p* < 0.05 versus D+Met (one-way ANOVA followed by Tukey’s post-hoc test). NC: normoglycaemic control, DC: diabetic control, D+MP: diabetic rats treated with Malaysian propolis, D+Met: diabetic rats treated with metformin, D+MP+Met: diabetic rats treated with Malaysian propolis + metformin, Nrf2: nuclear factor erythroid 2-related factor-2, SOD: superoxide dismutase, CAT: catalase, GPx: glutathione peroxidase, iNOS: inducible nitric oxide synthase.

The mRNA transcript levels of Nrf2, SOD and CAT were down-regulated (*p* < 0.05) in DC group compared to NC group, while GPx did not differ (*p* > 0.05) between DC and NC groups (Figure 4a–d). On the other hand, iNOS mRNA’s transcript level was up-regulated (*p* < 0.05) in DC group compared to NC group. Nrf2 mRNA’s transcript level was up-regulated (*p* < 0.05) in D+MP, D+Met and D+MP+Met groups, relative to DC group (Figure 4a). Furthermore, SOD and CAT’s mRNA transcript levels were up-regulated (*p* < 0.05) in D+MP and D+MP+Met groups, relative to DC group, with levels higher (*p* < 0.05) in D+MP+Met group relative to D+Met group (Figure 4b–c). GPx mRNA’s transcript level was highest in D+MP+Met group (*p* < 0.05), relative to DC, D+MP and D+Met groups (Figure 4d), while iNOS was down-regulated (*p* < 0.05) in all treatment groups relative to DC group (Figure 4e). Taken together, the mRNA transcript levels of Nrf2, SOD, CAT, GPx and iNOS genes in the treatment groups were comparable to NC group, except for CAT, which was lower (*p* < 0.05) in D+Met group relative to NC group.

### 3.5. Testicular Antioxidants and Oxidative Stress Status

Testicular SOD and CAT activities decreased (*p* < 0.05) in DC and D+Met groups relative to NC group, but increased (*p* < 0.05) in all treatment groups compared to DC group, with increase (*p* < 0.05) in D+MP+Met group compared to D+Met group (Table 2). Testicular GPx activity decreased (*p* < 0.05) in all the diabetic groups compared to NC group. However, GPx activity increased (*p* < 0.05) in all treatment groups compared to DC group, with an increase (*p* < 0.05) in D+MP+Met group compared to D+Met group (Table 2). Testicular GST activity decreased (*p* < 0.05) in DC and D+Met groups, while testicular GR activity decreased (*p* < 0.05) in DC, D+MP and D+Met groups relative to NC group, but increased (*p* < 0.05) in all treatment groups compared to DC group (Table 2). Among the treatment groups, GST activity was higher (*p* < 0.05) in the combined treatment group compared to D+Met group, while GR activity was higher (*p* < 0.05) in the combined treatment group relative to D+MP and D+Met groups. Both GST and GR activities in the combined treatment group were comparable to NC group (Table 2).

The testicular GSH level and TAC decreased (*p* < 0.05) in DC, D+MP and D+Met groups relative to NC group, but increased (*p* < 0.05) in all the treatment groups relative to DC group (Table 2). The GSH level in the combined treatment group was higher (*p* < 0.05) compared to D+Met group, while TAC was higher (*p* < 0.05) relative to D+MP and D+Met groups. Both GSH level and TAC in D+MP+Met group were comparable with NC group (Table 2).

The testicular TBARS level increased (*p* < 0.05) in DC, D+MP and D+Met groups, while testicular NO level increased (*p* < 0.05) in DC and D+Met groups compared to NC group (Table 2). TBARS and NO levels decreased (*p* < 0.05) in all treatment groups relative to DC group. Among the treatment groups, TBARS and NO levels decreased (*p* < 0.05) in D+MP and D+MP+Met groups relative to D+Met group. TBARS and NO levels in D+MP+Met group were comparable with NC group (Table 2).

### 3.6. Epididymal Antioxidants and Oxidative Stress Status

Epididymal SOD activity decreased (*p* < 0.05) in DC and D+Met groups, while CAT activity decreased (*p* < 0.05) in DC, D+MP and D+Met groups, compared to NC group. However, SOD and CAT activities increased (*p* < 0.05) in all treatment groups compared to DC group, with CAT activity in D+MP+Met group higher (*p* < 0.05) compared to D+Met group (Table 3). Epididymal GPx activity decreased (*p* < 0.05) in DC and D+Met groups, while GST and GR activities decreased (*p* < 0.05) in DC, D+MP and D+Met groups, relative to NC group. However, increases in GPx and GR activities were observed in all treatment groups (*p* < 0.05), while GST activity only increased (*p* < 0.05) in D+Met and D+MP+Met groups, relative to DC group (Table 3). The activities of GPx, GST and GR in the combined therapy group were higher (*p* < 0.05) relative to the monotherapy groups, and were comparable to NC group (Table 3).

The epididymal GSH level decreased (*p* < 0.05) in DC group, compared to NC group, but increased (*p* < 0.05) in all the treatment groups compared to DC group. GSH level in the treatment groups were comparable to NC group (Table 3). The epididymal TBARS level increased (*p* < 0.05) in DC, D+MP and D+Met groups relative to NC group. However, when compared to DC group, TBARS level decreased (*p* < 0.05) in all the treatment groups. Among the treatment groups, TBARS level decreased (*p* < 0.05) in the combined treatment group compared to the monotherapy groups (Table 3). Epididymal TAC decreased (*p* < 0.05) in all diabetic groups compared to NC group. Nevertheless, TAC increased (*p* < 0.05) in all treatment groups compared to DC group, with an increase (*p* < 0.05) in D+MP group relative to D+Met group, and an increase (*p* < 0.05) in D+MP+Met group relative to D+MP and D+Met groups (Table 3).

### 3.7. Testicular mRNA Transcript Levels and Immunoexpressions of Pro and Anti-Inflammatory Proteins

**Figure 5:**
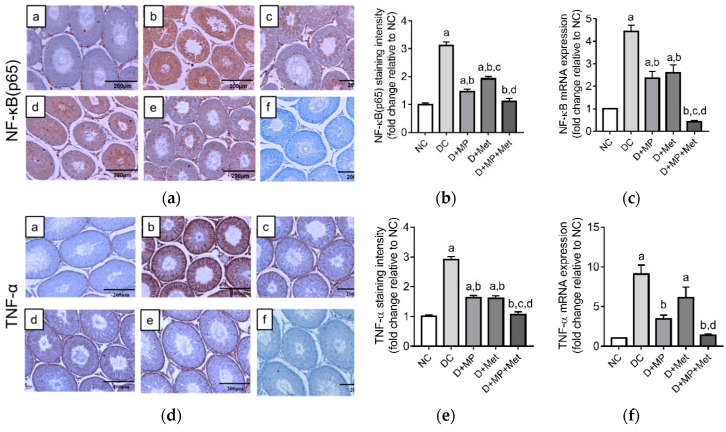
Expression of NF-κB (p65) protein (**a**,**b**) and mRNA (**c**); and the expression of TNF-α protein (**d**,**e**) and mRNA (**f**) in the testes of (**a**) NC, (**b**) DC, (**c**) D+MP, (**d**) D+Met and (**e**) D+MP+Met groups. For each biological replicate, a negative control was included, in which phosphate buffered saline was substituted for the primary antibody. A representative photomicrograph of the negative control is shown as (**f**) (magnification = ×100, scale bar = 200 µm). For quantitative data, values are mean ± SD, *n* = 8 (for staining intensity) and *n* = 6 (for mRNA expression). ^a^ *p* < 0.05 versus NC, ^b^ *p* < 0.05 versus DC, ^c^ *p* < 0.05 versus D+MP, ^d^ *p* < 0.05 versus D+Met (one-way ANOVA followed by Tukey’s post-hoc test). NC: normoglycaemic control, DC: diabetic control, D+MP: diabetic rats treated with Malaysian propolis, D+Met: diabetic rats treated with metformin, D+MP+Met: diabetic rats treated with Malaysian propolis + metformin, NF-κB: nuclear factor kappa B, TNF-α: tumour necrosis factor alpha.

The mRNA transcript levels and immunoexpressions of the pro-inflammatory markers; NF-κB and TNF-α, were up-regulated (*p* < 0.05) in DC group, relative to NC group (Figure 5a–f). Further, NF-κB and TNF-α’s mRNA transcript levels and immunoexpressions increased (*p* < 0.05) in the monotherapy groups, while TNF-α mRNA transcript level increased (*p* < 0.05) only in D+Met group, relative to NC group (Figure 5a–f). The mRNA transcript levels and immunoexpressions of NF-κB and TNF-α were down-regulated (*p* < 0.05) in all treatment groups, relative to DC group, with D+MP+Met group showing the best improvements (Figure 5a–f).

**Figure 6:**
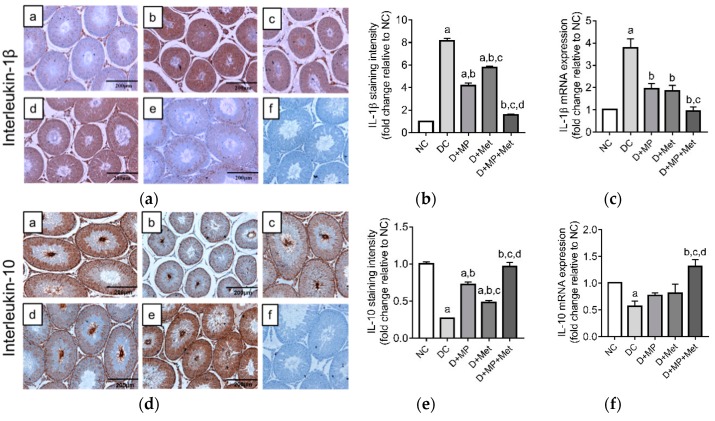
Expression of interleukin-1β protein (**a**,**b**) and mRNA (**c**), and interleukin-10 protein (**d**,**e**) and mRNA (**f**) in the testes of (**a**) NC, (**b**) DC, (**c**) D+MP, (**d**) D+Met and (**e**) D+MP+Met groups. For each biological replicate, a negative control was included in which phosphate buffered saline was substituted for the primary antibody. A representative photomicrograph of the negative control is shown as (**f**) (magnification = ×100, scale bar = 200 µm). For quantitative data, values are expressed as mean ± SD, *n* = 8 (for staining intensity) and *n* = 6 (for mRNA expression). ^a^ *p* < 0.05 versus NC, ^b^ *p* < 0.05 versus DC, ^c^ *p* < 0.05 versus D+MP, ^d^ *p* < 0.05 versus D+Met (one-way ANOVA followed by Tukey’s post-hoc test). NC: normoglycaemic control, DC: diabetic control, D+MP: diabetic rats treated with Malaysian propolis, D+Met: diabetic rats treated with metformin, D+MP+Met: diabetic rats treated with Malaysian propolis + metformin, IL: interleukin.

The mRNA transcript level and immunoexpression of pro-inflammatory IL-1β increased (*p* < 0.05), while anti-inflammatory IL-10 mRNA transcript level and immunoexpression decreased (*p* < 0.05) in DC group compared to NC group (Figure 6a–f). Treatment with MP, Met or their combination, down-regulated (*p* < 0.05) IL-1β’s mRNA level and immunoexpression, and up-regulated (*p* < 0.05) IL-10’s immunoexpression, relative to DC group. Only the combined therapy up-regulated (*p* < 0.05) IL-10’s mRNA transcript level relative to DC group (Figure 6f). Taken together, the combined therapy showed the best anti-inflammatory effect and was comparable to NC group.

### 3.8. Testicular Germ Cell Apoptosis and Proliferation

**Figure 7:**
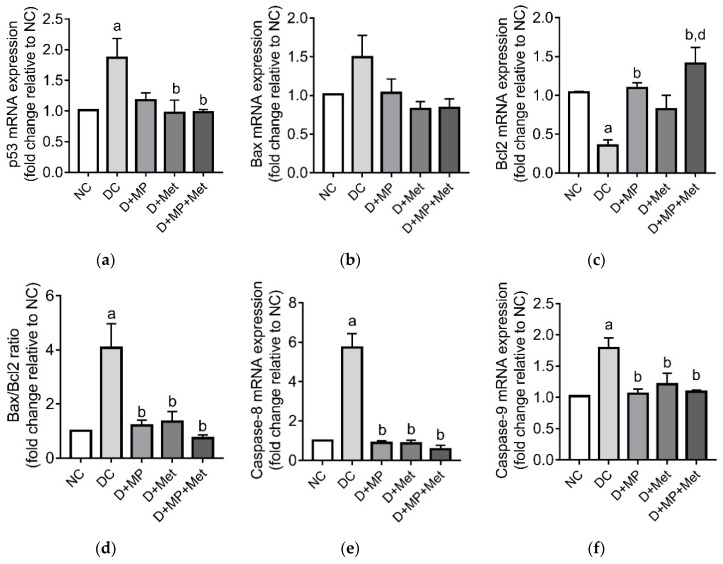
The effect of Malaysian propolis, metformin and their combination on testicular mRNA expressions of (**a**) p53, (**b**) Bax, (**c**) Bcl-2, (**d**) Bax/Bcl-2 ratio, (**e**) caspase-8 and (**f**) caspase-9, in diabetic rats. Values are mean ± SD, *n* = 6. ^a^ *p* < 0.05 versus NC, ^b^ *p* < 0.05 versus DC, ^d^ *p* < 0.05 versus D+Met (one-way ANOVA followed by Tukey’s post-hoc test). NC: normoglycaemic control, DC: diabetic control, D+MP: diabetic rats treated with Malaysian propolis, D+Met: diabetic rats treated with metformin, D+MP+Met: diabetic rats treated with Malaysian propolis + metformin, Bax: Bcl-2-associated-x-protein, Bcl-2: beta cell lymphoma-2, p53: tumour suppressor.

The mRNA transcript levels of selected apoptosis-related genes (p53, Bax, Bcl-2, caspase-8, caspase-9 and caspase-3) and protein level of the effector caspase (cleaved caspase-3) were assessed. The mRNA transcript levels of p53, Bax/Bcl-2, caspase-8 and caspase-9 genes were up-regulated (*p* < 0.05), while Bcl-2 gene was down-regulated (*p* < 0.05) in DC group relative to NC group (Figure 7a–f). In the treatment groups, p53’s mRNA transcript level decreased, but the levels were lower (*p* < 0.05) only in D+Met and D+MP+Met groups relative to DC group. The mRNA transcript level of Bax did not differ between the groups, even though it increased in DC group compared to NC group (Figure 7b). Bcl-2, on the other hand, was only up-regulated (*p* < 0.05) in D+MP and D+MP+Met groups, when compared to DC group (Figure 7c), while Bax/Bcl-2 ratio, caspase-8 and caspase-9 were down-regulated (*p* < 0.05) in all treatment groups relative to DC group (Figure 7d–f).

**Figure 8:**
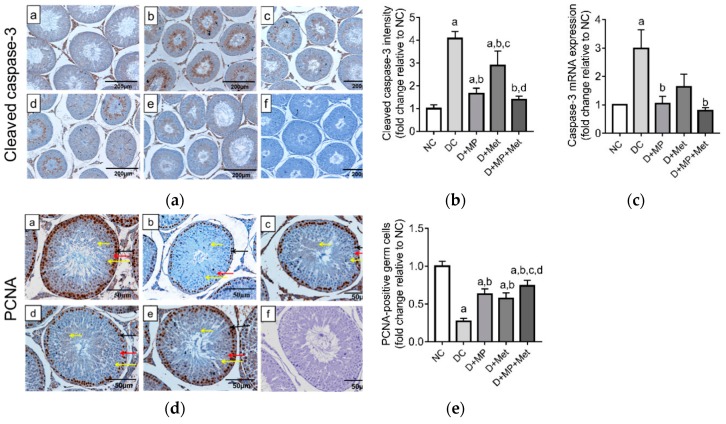
Expression of Caspase-3 protein (**a**,**b**) and mRNA levels (**c**), and proliferating cell nuclear antigen immunostaining (**d**,**e**) in the testes of (**a**) NC, (**b**) DC, (**c**) D+MP, (**d**) D+Met and (**e**) D+MP+Met groups. Representative photomicrograph of the negative control is shown as (**f**) (magnification = ×100, scale bar = 200 µm). For quantitative data, values are expressed as mean ± SD, *n* = 8 (for staining intensity) and *n* = 6 (for mRNA expression). ^a^ *p* < 0.05 versus NC, ^b^ *p* < 0.05 versus DC, ^c^ *p* < 0.05 versus D+MP, ^d^ *p* < 0.05 versus D+Met (one-way ANOVA followed by Tukey’s post-hoc test). NC: normoglycaemic control, DC: diabetic control, D+MP: diabetic rats treated with Malaysian propolis, D+Met: diabetic rats treated with metformin, D+MP+Met: diabetic rats treated with Malaysian propolis + metformin.

Caspase-3′s mRNA transcript level and immunoexpression increased in the testes of DC group compared to NC group (Figure 8a–c). Treatment with MP, Met or their combination decreased (*p* < 0.05) the expression of caspase-3 compared to DC group at both mRNA and protein levels, with comparable levels in D+MP+Met and NC groups (Figure 8a–c).

To examine testicular germ cell proliferation, PCNA immunoexpression was assessed. In the testes of NC group, PCNA-positive cells were mainly spermatogonia, spermatocytes and spermatids. PCNA-positive spermatogonia and spermatocytes were fewer in DC group compared to NC group, while in the treatment groups, the number of PCNA-positive germ cells were higher relative to DC group (Figure 8d). Quantitative data showed that PCNA-positive cells decreased (*p* < 0.05) in all the diabetic groups relative to NC group. However, PCNA-positive cells increased (*p* < 0.05) in all treatment groups relative to DC group, and increased (*p* < 0.05) in the combined treatment group relative to the monotherapy groups (Figure 8e).

## 4. Discussion

Several biological activities have been ascribed to propolis. In the present study, we explored its potential to abrogate oxidative stress, inflammation and apoptosis in the gonads of STZ-induced diabetic male rats. Further, we examined the possibility of MP serving as a complementary therapy with Met in the event that the monotherapies do not sufficiently counteract the negative effects of the disease. Consistent with our previous reports, MP demonstrated a significant anti-hyperglycaemic effect, which we previously attributed to pancreatic β-cell regeneration, increased insulin secretion, decreased hepatic gluconeogenesis and inhibition of α-glucosidase activity [20,26]. Worthy of noting is that the combined therapy (MP+Met) showed the best anti-hyperglycaemic effects, as demonstrated by a 4.87-fold decrease in final blood glucose level relative to DC group, while MP and Met only decreased the final blood glucose level by 2.32 and 2.98 fold, respectively. Further, the absolute and relative weights of the testes, epididymis, prostate and seminal vesicle decreased in the DC group in the present study, consistent with previous investigations [42,43,44]. Tissue wasting, organ scaring and loss of organ function are common in type 1 DM [45]. This may be attributable to a chronic lack of insulin, which triggers proteolysis in several tissues and organs, thus depleting structural proteins [46,47]. However, significant improvements were observed in the treatment groups. MP increased reproductive organs’ absolute and relative weights, while the combined treatment group showed the best improvements, with values comparable to NC group.

Seminiferous tubular atrophy and the depletion of germ cells were described as morphological indicators of spermatogenesis failure [48]. Earlier studies reported increased seminiferous tubule thickness, germ cell depletion and Sertoli cells’ vacuolization in diabetic rats, and in diabetic human testicular biopsies [49,50]. The histopathological findings in the untreated diabetic rats’ testes in the present study suggests a decline in spermatogenesis, and were consistent with the epididymal histopathology, which revealed large areas that were either devoid of spermatozoa or had decreased spermatozoan density. Furthermore, the epithelial height of the tubules in the epididymis increased in DC group, consistent with previous reports [51,52]. MP attenuated those negative changes in the testes and epididymis, comparably to metformin. Although there are no reports on the effects of propolis on testicular histology in a diabetic state, studies using Indian, Turkish and Egyptian propolis have demonstrated significant increases in testicular weight and seminiferous tubular diameter in rats after exposure to a heavy metal (cadmium) or chemotherapy (methotrexate, doxorubicin and mitomycin C) [53,54,55,56]. In those studies, counteracting oxidative stress was suggested as the possible mechanism of action of propolis when improving testes’ histopathological findings, as also observed in the present study.

Oxidative stress is generally considered as a major pathway for the development of diabetic complications [57]. It occurs when there is an imbalance between pro and anti-oxidants in favour of pro-oxidants. In DM, an increase in mitochondrial glucose oxidation, which results from hyperglycaemia, releases a large amount of ROS into the cytoplasm of cells, leading to an imbalance between pro and antioxidants in favour of pro-oxidants [9,58]. It has been reported that testicular germ cells are more susceptible to oxidative damage than somatic cells, because their plasma membrane contains more polyunsaturated fatty acids which are prone to oxidation by free radicals [59]. Therefore, excessive generation of ROS at a rate that outweighs the antioxidant defence system, as observed in DM, and the resulting oxidative stress, triggers germ cell death [6], thus impacting negatively on spermatogenesis and fertility potential [2]. Beyond the testes, oxidative stress may impact negatively on mature spermatozoa in the epididymis during storage. It is on that premise that markers of oxidative stress and antioxidant enzymes were assessed in both testes and epididymis in the present study.

The down-regulation of Nrf2 and some members of the antioxidant response element group (SOD, CAT and GPx) were seen in the testes of DC group, which are consistent with previous investigations [60,61,62]. Previous studies reported down-regulation of the other members of the antioxidant response elements (haem oxygenase-1 (HO-1) and NAD(P)H dehydrogenase(quinone)1 (NQO1)) controlled by Nrf2, in the testes of diabetic rats [60,62,63].

HO-1 plays a role in haem degradation, yielding bilirubin (which has antioxidant property) in the process, while NQO1 plays a role in reduction of ubiquinone and vitamin E derivatives to their antioxidant forms [64]. Nrf2 plays a critical role in the control of the expression and function of oxidative stress response genes, which is the reason it is referred to as the master regulator of redox status [65]. In fact, a previous study showed testicular oxidative stress and poor spermatogenesis in Nrf2 knockout mice, thus emphasising the crucial role of Nrf2 in maintaining redox status and spermatogenesis [66]. In the present study, by down-regulating Nrf2, the activities of antioxidant enzymes, particularly SOD, CAT, GPx, GST and GR, and the GSH level, decreased in the DC group, which is consistent with previous studies [44,67]. Similarly, epididymal antioxidant enzymes’ activities and GSH’s level decreased in DC group. From these results, we hypothesise that surviving spermatozoa from the testes may be prone to oxidative damage during storage in the epididymis.

The master regulator of redox status, Nrf2, is reported to be controlled by Kelch-like ECH-associated protein 1 (Keap1). Under normal conditions, Nrf2 is sequestered by Keap1 which results in its rapid degradation [68]. Inhibition of Keap1, therefore, prevents Nrf2 degradation, allowing the latter to be translocated into the nucleus, where it binds to the antioxidant response elements’ gene sites and up-regulates targeted gene expression (HO-1, NQO1, CAT and SOD) [68]. Though not assessed in the present study, previous studies have reported significant increases in Keap1 and cytosolic Nrf2 protein levels, and a decrease in the nuclear Nrf2 protein level in the testes of diabetic rats, clearly showing the inhibition of Nrf2 translocation to the nucleus by Keap1 [63]. Interestingly, polyphenols have been reported to up-regulate Nrf2 levels as part of their mechanisms of combating oxidative stress [63,69,70]. MP whole extract that was used in the present study was previously reported to contain high flavonoid and phenol contents [20]. Hence, the up-regulation of Nrf2 in the present study may be attributed to MP’s polyphenolic components.

Antioxidant enzymes play a major role in keeping the body free from oxidants. SOD is reported to catalyse the dismutation of O_2_^−^ to H_2_O_2_, which is then converted to H_2_O by CAT and/or GPx, without which the accumulated H_2_O_2_ will destroy lipid membranes and release large amounts of malondialdehyde (MDA) [71]. The action of GPx is mediated through reduced glutathione, and forms glutathione disulfide in the process. The latter is reduced to the sulfhydryl form GSH by GR [71]. The present study, therefore, shows that the SOD-CAT-GSH antioxidant defence system was significantly impaired in the testes and epididymis in the DC group, which may have resulted in the increased H_2_O_2_ level as previously reported [72].

Consistent with previous investigations [43,62], intra-testicular and epididymal TAC decreased significantly in the DC group of the present study. TAC is a measure of the synergistic interactions of the endogenous enzymatic and non-enzymatic antioxidant systems [73]. Decreased TAC, as seen in the DC group, is indicative of a significant decrease in the activity of the antioxidant defence system. This may have been orchestrated by increased intra-cellular ROS production and increased lipid peroxidation, as a previous study has reported increased intra-testicular total oxidative status in diabetic state [62]. Treatment with MP significantly increased antioxidant enzymes’ activities in both the testes and epididymis in the present study. These effects were comparable or better when compared with Met, with the best outcome following the combined treatment. Improvement in intra-testicular and epididymal antioxidant status may have played a significant role in improving testicular and epididymal cytoarchitecture, as observed in the treated diabetic groups, since oxidative stress inflicts structural damage. This assertion is supported by the fact that intra-testicular and epididymal TBARS, which is a product of lipid peroxidation, was significantly decreased following treatment with the various regimens.

Interestingly, MP demonstrated strong anti-oxidant activities in vitro (DPPH, FRAP and H_2_O_2_ scavenging activities), in addition to having high phenolic and flavonoid contents [20]. By scavenging H_2_O_2_, MP may have decreased its peroxidation effects on lipid membranes, thus decreasing TBARS’ level. The observed significant antioxidant activities may be attributable to the presence of gallic acid derivatives, coumaric acid derivatives, caffeic acid derivatives, ellagic acid and resveratrol in MP, which were previously identified by our group [31]. These findings are similar to those from previous studies, where propolis from Egypt, Turkey and India, were reported to decrease testicular MDA level and increase TAC after chemotherapy [53,56] or exposure to cadmium [54]. Furthermore, studies using MP obtained from *H. itama* demonstrated significant decreases in placental MDA and protein carbonyl levels, and an increase in TAC in diabetic dams [74].

Considering the above-discussed effects, it is plausible to hypothesise that MP may have improved testicular antioxidant status through three possible mechanisms: (i) a direct mechanism involving a synergy between MP and endogenous antioxidants, leading to scavenging of more ROS than only the endogenous antioxidants would do, thus sparing endogenous antioxidants and increasing their overall levels; (ii) up-regulation of Nrf2 and its downstream antioxidant response elements genes, thus up-regulating the mRNA levels and the activities of antioxidant enzymes; and (iii) an indirect mechanism involving a decrease in hyperglycaemia, thus decreasing the overall production of ROS, since the primary source of increased ROS production in DM is glucose auto-oxidation associated with hyperglycaemia [9]. The first two propositions are drawn from the fact that studies have demonstrated that the administration of antioxidants (quercetin, curcumin and resveratrol) significantly increases intra-testicular antioxidant activity, decreases lipid peroxidation and improves spermatogenesis without substantially decreasing blood glucose levels [57,63,75]. In fact, the fold changes in FBG level following treatment with quercetin and curcumin when compared to untreated diabetic rats were reported to be 1.09 and 1.13, respectively [57,75]. Since MP whole extract used in the present study demonstrated anti-hyperglycaemic effects, it is conceivable that the synergy of its phytoconstituents may have caused the significant decrease in FBG level, and improvement in antioxidant status, thus validating the third hypothesis above, which is centred on MP’s anti-hyperglycaemic effect.

The occurrence of oxidative damage in tissues is an indication of a likely occurrence of inflammation, since the duo are closely related, and to some extent, share common activation stimuli (ROS). The NF-κB-mediated inflammatory pathway is reportedly one of the targeted pathways of ROS [76]. Studies have reported that NO, which is released by vascular endothelial smooth muscle cells, plays a substantial role in the occurrence of both oxidative stress and inflammation [71]. In DM, NO is reported to be produced in high concentrations as a result of the up-regulation of iNOS [77]. The high circulating NO then interacts with other nitrogen and/or oxygen species, triggering nitrosative and/or oxidative stress [71]. NF-κB, a transcription factor, serves as a critical link between oxidative stress, inflammation and apoptosis. Upon activation by oxidative stress, NF-κB up-regulates iNOS’s level, leading to an increase in NO production. On the other hand, a high NO level can also trigger NF-κB up-regulation, thus initiating an inflammatory signalling cascade that, in turn, triggers the release of numerous inflammatory cytokines [71].

Consistent with our present study, previous studies have reported testicular inflammation in diabetic rats. Specifically, the activation of NF-κB, with increases in TNF-α, iNOS and IL-6 levels, have been reported in diabetic rats’ testes [6,7]. In the present study, the intra-testicular NO level increased notably in the DC group, in addition to increases in the mRNA and protein levels of NF-κB, iNOS, TNF-α and IL-1β, and a decrease in IL-10. Significant improvements were seen in the treated diabetic groups. Particularly, D+MP+Met group was comparable to NC group, while MP treatment yielded better results when compared to Met. The combined therapy, thus, appears to offer better protection from DM-induced inflammation. This effect may not be unconnected to the near-normal blood glucose level recorded in this group, since inflammation in the gonad is orchestrated by persistent hyperglycaemia and oxidative stress. The anti-inflammatory effect of MP in the testes is consistent with previous report from our group, where MP significantly decreased NF-κB (p65), TNF-α and IL-1β protein expressions, and increased IL-10 protein expression in the pancreases and livers of diabetic rats [20,26].

Studies have reported increased testicular germ cell apoptosis in DM, which occurs as a result of the interaction between oxidative stress and inflammation. In the present study, the up-regulation of pro-apoptotic p53, Bax/Bcl-2 ratio, caspase-8 and caspase-9, are indicative of the contributions from both intrinsic and extrinsic apoptotic signalling, both of which lead to the activation of caspase-3, as previously reported [8,78,79]. Following a build-up of oxidants, p53 up-regulates pro-apoptotic Bax, releasing cytochrome-c which drives the intrinsic apoptotic pathway, and subsequent activation of the executioner, caspase-3 [80,81]. Although a TUNEL assay was not carried out on the testes in the present study, up-regulation of both mRNA and protein levels of caspase-3 in this study may suggest increased testicular germ cell apoptosis. Further, there were less PCNA-positive germ cells in the DC group, which implies poor cell proliferation, and supports the idea that most of the germ cells could have undergone apoptosis. Previous studies have reported an increase in TUNEL-positive germ cells in the testes of diabetic rats [57,60], thus corroborating our hypothesised increase in apoptosis in diabetic rats’ testes in the present study. Interestingly, MP treatment decreased apoptosis in the testes, as seen with the significant decreases in the mRNA and protein levels of caspase-3. The result of the present study is consistent with previous studies using Egyptian and Turkish propolis, which reported decreased testicular germ cell apoptosis after exposure to chemotherapy [54,55]. The inhibitory effects of MP on apoptosis in the present study may be associated with decreases in oxidative stress and inflammation.

Overall, MP demonstrated comparable effects with Met, and better effects in some regard. Whether or not MP could substitute Met or any other anti-hyperglycaemic medication, cannot be inferred in the present study. However, treatment with MP+Met offered a better protection against DM-induced oxidative stress, inflammation and apoptosis in the gonad, relative to MP and Met monotherapies. This maybe attributable to the fact that Met decreases hepatic gluconeogenesis, improves the sensitivity of cells to circulating insulin and improves glucose uptake [82], while MP acts on the pancreas to increase insulin level, and decreases hepatic gluconeogenesis, in addition to having inherent antioxidant potential [20,26]. The combination proved more beneficial, as seen in the present study, and therefore, is a promising therapy that requires further mechanistic investigations.

## 5. Conclusions

Malaysian propolis mitigated testicular and epididymal oxidative stress, and down-regulated NF-κB-mediated inflammation and apoptosis in diabetic rat testes. Those beneficial effects were potentiated following its co-administration with metformin, suggesting a possible complementary effect. Therefore, MP or its combination with Met, may improve the fertility potential of males with DM, since they decreased diabetic complications in the testes and epididymis.

## Figures and Tables

**Table 1:** Weights and relative weights of reproductive organs in all experimental groups.

Parameter		NC	DC	D+MP	D+Met	D+MP+Met
Testes	AW (g)	3.64 ± 0.39	1.49 ± 0.36 ^a^	3.31 ± 0.40 ^b^	2.86 ± 0.20 ^a,b^	3.37 ± 0.37 ^b,d^
	RW (%)	0.99 ± 0.18	0.75 ± 0.18 ^a^	1.03 ± 0.15 ^b^	0.91 ± 0.11	0.97 ± 0.16
Epididymis	AW (g)	1.30 ± 0.17	0.62 ± 0.14 ^a^	1.05 ± 0.04 ^a,b^	0.93 ± 0.16 ^a,b^	1.17 ± 0.10 ^b,d^
	RW (%)	0.35 ± 0.06	0.32 ± 0.09	0.33 ± 0.04	0.30 ± 0.06	0.34 ± 0.06
Prostate	AW (g)	0.59 ± 0.13	0.21 ± 0.05 ^a^	0.52 ± 0.11 ^b^	0.36 ± 0.08 ^a^	0.65 ± 0.21 ^b,d^
	RW (%)	0.17 ± 0.04	0.11 ± 0.03 ^a^	0.16 ± 0.04 ^b^	0.12 ± 0.03 ^a^	0.18 ± 0.05 ^b,d^
Seminal vesicle	AW (g)	1.92 ± 0.27	0.34 ± 0.11 ^a^	1.25 ± 0.34 ^a,b^	1.01 ± 0.26 ^a,b^	1.92 ± 0.28 ^b,c,d^
	RW (%)	0.52 ± 0.10	0.17 ± 0.06 ^a^	0.39 ± 0.11 ^a,b^	0.32 ± 0.08 ^a,b^	0.55 ± 0.07 ^b,c,d^

Values are mean ± SD, *n* = 8. ^a^ *p* < 0.05 versus NC, ^b^ *p* < 0.05 versus DC, ^c^ *p* < 0.05 versus D+MP, ^d^ *p* < 0.05 versus D+Met (one-way ANOVA followed by Tukey’s post-hoc test). AW: absolute weight, RW: relative weight, NC: normoglycaemic control, DC: diabetic control, D+MP: diabetic rats treated with Malaysian propolis, D+Met: diabetic rats treated with metformin, D+MP+Met: diabetic rats treated with Malaysian propolis + metformin.

**Table 2:** Testicular antioxidant enzymes, oxidative stress markers and total antioxidant capacity.

Groups	NC	DC	D+MP	D+Met	D+MP+Met
SOD activity (unit/mg protein)	2.21 ± 0.08	1.32 ± 0.08 ^a^	2.06 ± 0.19 ^b^	1.98 ± 0.12 ^a,b^	2.23 ± 0.13 ^b,d^
CAT activity (unit/mg protein)	38.88 ± 4.85	11.51 ± 2.98 ^a^	32.36 ± 4.42 ^b^	27.24 ± 3.53 ^a,b^	38.49 ± 7.24 ^b,d^
GPx activity (unit/mg protein)	35.83 ± 3.17	10.46 ± 1.84 ^a^	27.51 ± 2.71 ^a,b^	22.87 ± 3.21 ^a,b^	30.41 ± 2.39 ^a,b,d^
GST activity (unit/mg protein)	167.90 ± 7.52	119.50 ± 4.84 ^a^	161.90 ± 5.16 ^b^	156.30 ± 5.23 ^a,b^	167.50 ± 4.24 ^b,d^
GR activity (unit/mg protein)	23.52 ± 2.36	11.01 ± 1.38 ^a^	17.76 ± 2.04 ^a,b^	16.19 ± 1.01 ^a,b^	20.85 ± 2.31 ^b,c,d^
GSH level (nmol GSH Eq./mg protein)	2.96 ± 0.48	1.15 ± 0.19 ^a^	2.35 ± 0.28 ^a,b^	2.01 ± 0.24 ^a,b^	2.61 ± 0.41 ^b,d^
TAC (nmol uric acid Eq./mg protein)	173.70 ± 8.60	77.46 ± 9.37 ^a^	158.00 ± 9.58 ^a,b^	141.50 ± 9.50 ^a,b,c^	183.50 ± 4.80 ^b,c,d^
TBARS level (nmol/mg protein)	1.03 ± 0.15	3.53 ± 0.26 ^a^	1.57 ± 0.29 ^a,b^	2.40 ± 0.31 ^a,b,c^	1.38 ± 0.27 ^b,d^
Nitric oxide level (µmol/g tissue)	2.66 ± 0.93	14.03 ± 2.41 ^a^	3.62 ± 0.98 ^b^	6.68 ± 1.57 ^a,b,c^	3.62 ± 0.52 ^b,d^

Values are mean ± SD, *n* = 8. ^a^ *p* < 0.05 versus NC, ^b^ *p* < 0.05 versus DC, ^c^ *p* < 0.05 versus D+MP, ^d^ *p* < 0.05 versus D+Met (one-way ANOVA followed by Tukey’s post-hoc test). SOD: superoxide dismutase, CAT: catalase, GPx: glutathione peroxidase, GR: glutathione reductase, GST: glutathione-S-transferase, GSH: total glutathione, TAC: total antioxidant capacity, TBARS: thiobarbituric acid reactive substance, NC: normoglycaemic control, DC: diabetic control, D+MP: diabetic rats treated with Malaysian propolis, D+Met: diabetic rats treated with metformin, D+MP+Met: diabetic rats treated with Malaysian propolis + metformin.

**Table 3:** Epididymal antioxidants, TBARS level and total antioxidant capacity.

Groups	NC	DC	D+MP	D+Met	D+MP+Met
SOD activity (unit/mg protein)	2.89 ± 0.38	1.22 ± 0.26 ^a^	2.43 ± 0.22 ^b^	2.21 ± 0.31 ^a,b^	2.76 ± 0.40 ^b,d^
CAT activity (unit/mg protein)	28.32 ± 3.02	15.18 ± 3.00 ^a^	24.24 ± 2.89 ^a,b^	23.27 ± 2.50 ^a,b^	26.46 ± 2.03 ^b^
GPx activity (unit/mg protein)	34.08 ± 4.89	16.77 ± 3.06 ^a^	30.06 ± 3.34 ^b^	27.77 ± 2.27 ^a,b^	35.33 ± 3.22 ^b,c,d^
GST activity (unit/mg protein)	140.40 ± 13.57	91.15 ± 2.77 ^a^	104.80 ± 13.26 ^a^	108.50 ± 6.26 ^a,b^	127.00 ± 9.02 ^b,c,d^
GR activity (unit/mg protein)	22.29 ± 1.39	10.31 ± 1.29 ^a^	18.41 ± 1.26 ^a,b^	16.64 ± 1.59 ^a,b^	18.75 ± 1.41 ^b,c,d^
GSH level (nmol GSH Eq./mg protein)	2.28 ± 0.32	0.91 ± 0.12 ^a^	2.01 ± 0.25 ^b^	1.93 ± 0.25 ^b^	2.15 ± 0.29 ^b^
TBARS level (nmol/mg protein)	1.61 ± 0.19	7.05 ± 0.74 ^a^	3.73 ± 0.59 ^a,b^	4.45 ± 0.80 ^a,b^	2.32 ± 0.47 ^b,c,d^
TAC (nmol uric acid Eq/mg protein)	249.60 ± 17.79	95.57 ± 11.15 ^a^	197.40 ± 17.19 ^a,b^	153.20 ± 18.56 ^a,b,c^	224.10 ± 15.52 ^a,b,c,d^

Values are mean ± SD, *n* = 8. ^a^ *p* < 0.05 versus NC, ^b^ *p* < 0.05 versus DC, ^c^ *p* < 0.05 versus D+MP, ^d^ *p* < 0.05 versus D+Met (one-way ANOVA followed by Tukey’s post-hoc test). SOD: superoxide dismutase, CAT: catalase, GPx: glutathione peroxidase, GR: glutathione reductase, GST: glutathione-S-transferase, GSH: total glutathione, TAC: total antioxidant capacity, TBARS: thiobarbituric acid reactive substance, NC: normoglycaemic control, DC: diabetic control, D+MP: diabetic rats treated with Malaysian propolis, D+Met: diabetic rats treated with metformin, D+MP+Met: diabetic rats treated with Malaysian propolis + metformin.

## Supplementary Materials

The following are available online at https://www.mdpi.com/2076-3921/8/10/465/s1: Table S1: Oligonucleotides used for PCR amplification of antioxidant, inflammation and apoptosis-related genes.
