# Supplementary material for: Oxidative Stress, NF-κB-Mediated Inflammation and Apoptosis in the Testes of Streptozotocin–Induced Diabetic Rats: Combined Protective Effects of Malaysian Propolis and Metformin
Source: Antioxidants (Basel). 2019 Oct 9;8(10):465. doi: 10.3390/antiox8100465 (PMC6826571; doi:10.3390/antiox8100465)
Supplement: Supplementary file 1 [file antioxidants-08-00465-s001.pdf]

Supplementary Table 1. Oligonucleotides used for PCR amplification of antioxidant, inflammation and apoptosis-related genes

Gene	Accession Number	Primer Sequence		Amplicon Size (bp)
Nrf2	NM_031789.1	FCAGGTTGCCCACATTCCCAA	RATATCCAGGGCAAGCGACTCAT	110
SOD	X05634.1	FCGAGCATGGGTTCCATGTC	RCTGGACCGCCATGTTTCTTAG	101
CAT	NM_012520.2	FACAACTCCCAGAAGCCTAAGAATG	RGCTTTTCCCTTGGCAGCTATG	76
GPx	NM_030826.4	FGGAGAATGGCAAGAATGAAGA	RCCGCAGGAAGGTAAAGAG	139
NF-kB	NM_199267.2	FCGCGGGGACTATGACTTGAA	RAGTTCCGGTTTACTCGGCAG	163
TNF-α	NM_012675.3	FACTGAACTTCGGGGTGATCG	RGCTTGGTGGTTTGCTACGAC	153
iNOS	XM_006246949.3	FCAGCCCTCAGAGTACAACGAT	RCAGCAGGCACACGCAATGAT	91
IL-1β	NM_031512.2	FGACTTCACCATGGAACCCGT	RGGAGACTGCCCATTCTCGAC	104
IL-10	NM_012854.2	FTTGAACCACCCGGCATCTAC	RCCAAGGAGTTGCTCCCGTTA	91
p53	NG_005120.4	FCTACTAAGGTCGTGAGACGCTGCC	RTCAGCATACAGGTTTCCTTCCACC	106
Bax	U49729.1	FCGCGTGGTTGCCCTCTTCTACTTT	RCAAGCAGCCGCTCACGGAGGA	124
Bcl-2	NM_016993.1	FATCGCTCTGTGGATGACTGAGTAC	RAGAGACAGCCAGGAGAAATCAAAC	134
Caspase-8	NM_022277.1	FGTTCTCTCAGTTGCCTTTCTCC	RGGCCAGTCCGCCAAAGTTTA	90
Caspase-9	NM_031632	FCTGAGCCAGATGCTGTCCCATA	RCCAAGGTCTCGATGTACCAGGAA	168
Caspase-3	NM_012922	FAAGATACCAGTGGAGGCCGACTTC	RGGGAGAAGGACTCAAATTCCGTGG	199
GAPDH	NM_017008	FTCACCACCATGGAGAAGGC	RGCTAAGCAGTTGGTGGTGCA	169

Bax: Bcl-2-associated X protein, Bcl-2: beta cell lymphoma-2, Nrf2: nuclear factor erythroid 2–related factor 2, SOD: superoxide dismutase, CAT: catalase, GPx: glutathione peroxidase, NF-kB: nuclear factor kappa B, TNF: tumour necrosis factor, iNOS: inducible nitric oxide synthase, IL: interleukin, GAPDH: glyceraldehyde-3-phosphate dehydrogenase, p53: tumour protein.
